# The Effect of Various Combinations of Peripheral Nerve Blocks on Postoperative Pain in Laparoscopic Cholecystectomy: A Comparative Prospective Study

**DOI:** 10.1155/2023/8864012

**Published:** 2023-11-23

**Authors:** Zoya Haitov Ben Zikri, Maryna Volis, Andrei Mazur, Tatjana Orlova, Hana Alon, Sara Bar Yehuda, Vladislav Gofman

**Affiliations:** Anesthesiology Department, Shamir Medical Center, Affiliated to Sackler Faculty of Medicine, Tel Aviv University, Zerifin 70300, Israel

## Abstract

**Objectives:**

Most patients who undergo laparoscopic cholecystectomy (LC) experience moderate to severe pain in the first 24 hours after surgery. The transversus abdominal plane (TAP) is currently used for post-LC analgesia. Posterior, subcostal, or rectus sheath TAP blocks are the conventional approaches used. The aim of the current study was to compare the efficacy of combinations of various peripheral blocks on pain intensity and the use of pain killers, shortly after LC.

**Methods:**

This was a prospective, double-blind study, in which 200 patients who were about to undergo a LC procedure were recruited and randomized into 4 groups: patients receiving one of the following: TAP block alone, subcostal Tap block alone, subcostal TAP block with a TAP block, or subcostal TAP with a rectus sheath block. The intensity of pain (VAS score) and the use of painkillers were monitored in the recovery unit and in the department for up to 24 hours after surgery.

**Results:**

Pain levels decreased with time from 3.6 ± 3.2 at 30 minutes to 0.9 ± 2.0 at 24 hours after the surgery. Nevertheless, no difference between the various block types groups was noted. The percentage of patients who consumed analgesic medications decreased over time, from 83% at 30 to 21% at 24 hours after surgery. The mean/median number of medications consumed by each of the patients was lower among the patients who received a combination of 2 blocks compared to those who received a single one (mean/median of 2.7/3 and 2.8/3 for the TAP or subcostal TAP blocks, respectively; 2.5/2 and 2.3/2 for the subcostal TAP + TAP or subcostal TAP + rectus sheath blocks, respectively).

**Conclusion:**

A combination of peripheral nerve blocks reduced the use of analgesic consumption during the 24 hours after LC surgery, compared to standalone blocks.

## 1. Introduction

Laparoscopy is a minimally invasive technique that is most frequently performed during upper abdominal surgery. The laparoscopy technique is considered to be superior to open surgery due to lower postoperative pain and early recovery [1]. This procedure is the gold standard treatment modality for gallbladder disorders, such as cholelithiasis and cholecystitis [2, 3]. Despite the advantages of the laparoscopic approach regarding pain control, most patients who undergo laparoscopic cholecystectomy (LC) experience moderate to severe pain in the first 24 hours after surgery, with port sites being the most painful [4]. Three kinds of pain have been described to occur after LC: somatic pain, caused by the skin incision; a deep intra-abdominal visceral pain due to the trauma of gallbladder resection and diaphragmatic irritation by the CO_2_ pneumoperitoneum; and shoulder pain, also due to the CO_2_ pneumoperitoneum [5, 6]. The intensity of the postoperative pain reported is very variable [7].

Postoperative pain is among the primary causes of reduction in respiratory function after upper abdominal surgery [8]. Efficient pain control after surgery results in rapid mobility and a decreased postoperative complication rate and facilitates early hospital discharge. Therefore, many efforts have been devoted to optimizing postoperative pain control. Usually, the analgesic regimen upon LC includes opioids, paracetamol, conventional NSAID/COX2 selective inhibitors, epidural analgesia, intraperitoneal injection of local anesthetics, or low-pressure pneumoperitoneum. However, the side effects of the administered medications (such as excessive sedation, nausea and vomiting, dizziness, pruritus, and respiratory depression), the potential risk of dural puncture, infection, and epidural hematoma, as well as muscle weakness, insufficient pain control, and short duration of the analgesia, must be considered [9–11]. The transversus abdominal plane (TAP) block which was introduced for the first time in 2001 by Rafi [12] is currently used for postoperative analgesia during laparoscopic abdominal surgery [13]. In this procedure, a local anesthesia solution is injected into the plane between the obliquus internus and transversus abdominis muscles, exiting the T6 to L1 spinal roots [12, 14]. The TAP block is safe; it reduces or eliminates the need for analgesics and has fewer side effects such as postoperative nausea and vomiting [15]. Thus, the TAP blocks are considered a good clinical tool for analgesia after abdominal surgery for pain relief. Indeed, TAP blocks are used in a number of abdominal procedures including hysterectomy, cesarean section, cholecystectomy, colectomy, hernia repair, and prostatectomy [12, 16–18]. Jeffrey et al. compared opioid requirements after surgery among patients who underwent LC with (*n* = 100) or without TAP (*n* = 100) in a retrospective cohort study. The mean postoperative opioid consumption was significantly lower among the patients who had LC with TAP compared to those who did not receive TAP (12.1 vs. 20.4 oral morphine respectively, *P* < 0.001). Patients who had LC with TAP reported at the follow-up visit that they used less of their prescribed opioids in comparison to patients who underwent LC without TAP (*P* < 0.001) [19].

Although the conventional method of the TAP block is the posterior approach, the subcostal TAP block has been recently reported to induce an effective analgesia for the upper abdominal incisions [20]. By this technique, a local anesthetic is injected into the TAP lateral to the linea semilunaris, inferior and parallel to the costal margin [21]. It was found that the subcostal TAP technique can block the T7 to T12 nerves [22]. Hence, the subcostal TAP block provides analgesia for surgery on the upper abdominal wall, thus suitable for abdominal surgery [21, 23]. In a prospective, randomized, controlled trial, 110 patients who underwent LC were equally randomized to one of three groups receiving TAP, subcostal TAP, and no TAP (the later serving as a control group). Both TAP and subcostal TAP groups were significantly and equally effective in inducing pain relief and reducing analgesic requirements compared to the no TAP control group [24].

An additional technique to provide analgesia to the anteromedial abdominal wall is the rectus sheath block, first described in 1899 by Schleich. This technique targets the area between the rectus abdominis muscle and the posterior rectus muscle sheath. It blocks the anterior cutaneous branches of the intercostal nerves (at T7-T11) and therefore is well suited for postoperative analgesia for midline abdominal incisions [25, 26]. Hamid et al. conducted a meta-analysis of 9 trials (698 patients) comparing the use of rectus sheath block to no regional anesthetic technique among laparoscopic surgery patients. The patients in the rectus sheath block group had significantly lower resting and active pain scores at 2 h after surgery. In addition, the use of opioid use at 24 h after surgery decreased significantly in the rectus sheath block group compared to the no regional anesthetic technique (SMD−1.34 (95% CI −2.20 to −0.49)). Consequently, the treatment with the rectus sheath block group reduced the risk of opioid-related side effects (OR 0.38 (95% CI 0.16 to 0.89)) [27].

The aim of the current study was to compare the efficacy of combinations of various peripheral blocks on pain intensity and the use of pain killers, shortly after LC. Tap and subcostal TAP as standalone blocks were compared to the combination of subcostal TAP + TAP blocks and subcostal TAP + rectus sheath blocks.

## 2. Methods

### 2.1. Study Design

This was a prospective, double-blind study, in which 200 patients who were about to undergo a LC procedure were recruited. The study was approved by the Institutional Review Board (study approval no. 0238-19-ASF) and registered at the My Trial database of the Israeli Ministry of Health (registration number MOH_2019-12-12_008555). All patients signed an informed consent form. The patients were randomized into 4 groups: patients receiving one of the following a TAP block alone, a subcostal Tap block alone, a subcostal TAP block with a TAP block or a subcostal TAP with a rectus sheath block. All those peripheral blocks were injected after the administration of the general anesthesia, before any surgical incision was performed. No sedatives were administered to the patients before the surgery procedure.

The anesthesia was performed by a specialist anesthesiologist with over 10 years' experience who had over two years practice in the administration of the tested peripheral blocks.

### 2.2. Patients

Male and female patients aged 18–70 years who were about to undergo a LC procedure participated in the study. Patients with any sensitivity to the anesthetic or with BMI ≥40 kg/m^2^ were excluded from the study.

### 2.3. Anesthesia Process

In the operating room, the patient was attached to a standard monitor, recording electroencephalogram, noninvasive blood pressure, and saturation.

Preoxygenation was performed for 4 minutes with 2 mg/kg fentanyl administered intravenously (IV), 2 minutes of 2 mg/kg propofol and 0.5 mg/kg rocuronium. The patient was then intubated and connected to an anesthesia machine. The anesthesia was maintained by isoflurane 1 MAC, muscle relaxants according to monitoring (3-4 mg/kg fentanyl). Additional monitoring of end-tidal carbon dioxide, body temperature, anesthetic gases, and breathing parameters was conducted after intubation. The administration of the various peripheral blocks was done after the patient was connected to the anesthesia machine. The blocks included 0.25% Marcaine (30 ml) and 2 mg dexamethasone. After surgery and extubation, the patient was transferred to the recovery unit.

### 2.4. Monitoring the Intensity of Pain and the Use of Painkillers

The intensity of pain and the use of painkillers were monitored in the recovery unit and in the department for up to 24 hours after surgery. Time points of monitoring were 30 and 60 minutes, 6, 12, and 24 hours after surgery.

Pain intensity was monitored by the Visual Analogue Scale (VAS).

Painkillers included NSAIDs and opioids. The consumption of the following painkillers was monitored: paracetamol (1 gr.), tramal (100–200 mg.), morphine (10 mg.), IV dipyrone (1 gr.), ketorolac (20–60 mg.), pethidine (50–100 mg.), and diclofenac (75 mg.).

### 2.5. Statistical Analysis

The Pearson correlation coefficient was calculated for the correlation between age and pain levels; *χ*^2^ tests were used to identify the relationship between gender and categories of pain levels as well as drug types in each block and time point. One-way ANOVA was used to compare the mean number of drugs of the various blocks.

A mixed model analysis of variance calculated differences in the decrease in pain levels over time within each block and between the different blocks. P < 0.05 was considered significant.

## 3. Results

One hundred and eighty-nine patients were recruited for this study and were randomized equally into the 4 study groups. Most of the patients were females (66%). The mean age of the patients was 53.02 ± 17.1 years (range 18–80 years).

### 3.1. Pain

**Figure 1:**
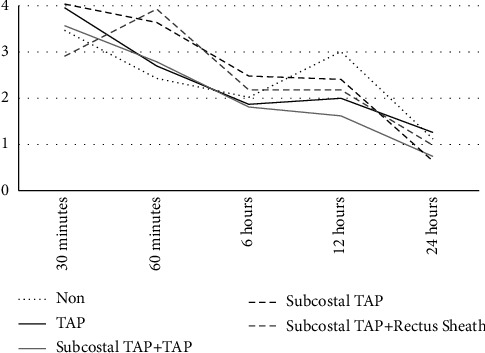
Comparison of pain levels at any time point between the groups.

As reported by the patients, pain levels decreased with time from 3.6 ± 3.2 at 30 minutes to 0.9 ± 2.0 at 24 hours after the surgery. A decrease in the levels of pain within time was also noted in all the block types groups, but no statistical significance between the groups was noted (Tap block: from 3.8 ± 3.1 at 30 minutes to 1.1 ± 2.2 at 24 hours after the surgery; subcostal TAP block: from 4.0 ± 3.2 at 30 minutes to 0.6 ± 1.8 at 24 hours after the surgery; subcostal TAP + TAP blocks: from 3.6 ± 3.3 at 30 minutes to 0.7 ± 1.9 at 24 hours after the surgery; and subcostal TAP + rectus sheath blocks: from 2.9 ± 3.2 at 30 minutes to 1.0 ± 2.1 at 24 hours after surgery (Figure 1 and Table 1).

A mixed model analysis of variances also revealed that there was a clear difference in the decrease in pain levels over time (*F*(4.0535) = 57.843 and P < 0.001). Nevertheless, no difference between the various block types groups was noted (*F*(12.590) = 7.118 and P = 0.420).

A 2-tailed Pearson correlation coefficient test found a statistically significant relationship between age and level of pain at the time points of 30 minutes and 24 hours; as age increases, the level of pain decreases (Pv = 0.002 and 0.017 and *r* −0.222 and −0.176, respectively). *χ*2 tests showed that there was no correlation between the gender and the pain level at any time point.

### 3.2. Medication

The percentage of patients who consumed analgesic medications decreased over time, from 83% to 71% at 30 and 60 minutes to 39% and 43% at 6 and 12 hours, down to 21% at 24 hours after surgery. Overall, 82% of the patients consumed paracetamol, 62% morphine, 54% dipyrone IV, and 49% tramal. The medications at 30 and 60 minutes after surgery were paracetamol and morphine (31% and 41% of the patients took those medications 30 minutes after surgery and 20% and 25% of the patients used them at 60 minutes after surgery, respectively). Six hours after the surgery, tramal, dipyrone IV, and paracetamol were the medications most administered (used by 13%, 1%, and 11% of the patients, respectively). Twelve hours after surgery, tramal and dipyrone IV were the main administered medications (consumed by 14% and 16% of the patients, respectively). Twenty-four hours after surgery, dipyrone IV was the most dispensed medication (administered to 8% of the patients) (Table 2).

Interestingly, the mean/median number of medications consumed by each of the patients was lower among the patients who received a combination of 2 blocks compared to those who received a single one (mean/median of 2.7/3 and 2.8/3 for the TAP or subcostal TAP blocks, respectively; 2.5/2 and 2.3/2 for the subcostal TAP + TAP or subcostal TAP + rectus sheath blocks, respectively). An independent-samples median test revealed that the difference in the mean/median between subcostal TAP single block treatment and the combined treatment of subcostal TAP + TAP was statistically significant with a P value of 0.025 (Table 3).

**Figure 2:**
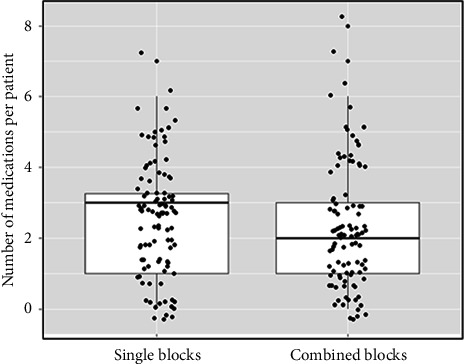
Comparison of mean/median number of medications consumed by each of the patients treated with a single block versus combined blocks treatments.

In addition, an independent-samples Kolmogorov–Smirnov test demonstrated that when pooling data of the number of medications consumed by each patient from the single block treatments and comparing it to the pooled data of the combined blocks treatments, a statistically significant difference was noted. Moreover, fewer medications per patient were administered to the patients receiving a combination of blocks compared to those who were treated with a single block (mean/median of 2.73/3 in the single treatment vs. 2.4/2 in the combined treatment, Pv = 0.024) (Table 4 and Figure 2).

It is important to note that during the 24 hours in which the patients were monitored, no side effects were reported, neither in the single block nor in the combined blocks treated patients.

## 4. Discussion

Post-LC pain is multifactorial, and therefore, multimodal analgesia has been suggested for its treatment. There are several methods for postoperative pain management, including peripheral nerve block techniques, which have received much attention recently due to their positive effect in reducing postoperative pain and good tolerance [4]. TAP, subcostal TAP, and rectus sheath blocks are some of the methods that have been recommended for the management of postoperative pain, especially for somatic pain control of the abdomen.

There are studies that have demonstrated the efficacy of each of the above blocks in reducing LC postoperative pain scores and lowering the consumption of painkiller. For example, Kadam et al. conducted a study aimed to evaluated postoperative pain and analgesic use during the first day after LC surgery, compared to local anesthetics and the TAP block. There were no significant differences in the postoperative pain scores between the local anesthetic and TAP block groups (*P* = 0.31) However, fentanyl consumption in the recovery room was significantly lower in the TAP group (*P* = 0.0079) [28]. In an additional study, 100 LC patients were randomized into two groups of TAP block and port-site infiltration. The median VAS at 3, 6, and 24 hours postoperation were significantly lower in the TAP group compared to the port-site infiltration group (*P* ≤ 0.001) [29]. Pain severity and opioid requirements, 1, 4, and 8 hours after LC, were compared between patients who received subcostal TAP and those who were treated by a port-site infiltration of local anesthetics. The subcostal TAP induced a significant reduction in VAS values and a reduction of >35% in opioid consumption compared to the group that received local port-site infiltration [30]. In a retrospective study conducted by Tekeli et al., 515 patients who underwent an LC procedure were divided into two groups: those who received bilateral subcostal TAP block after anesthesia induction and those who were treated by a dual IV analgesic 30 minutes before the end of the operation. Postoperative VAS pain scores at 0, 2, 4, and 6 hours and the rate of analgesic medication use were significantly lower in the group that received the subcostal TAP block compared to those who were treated only with an additional analgesic. Interestingly, this effect was reversed at 12 and 24 hours postoperatively, since the intensity of the pain was significantly higher in the subcostal TAP block group. The authors suggested that the relatively low VAS pain scores reported at 12- and 24-hours were due to the fact that shortly after surgery (up to 6 hours), the patients in this group reported high levels of pain and were treated with additional analgesics, which induced a lasting pain relief effect for the next few hours [15]. In a systematic literature search aimed to identify trials comparing the rectus sheath block with a control group in laparoscopic surgery, 9 trials with 698 patients were screened. In the rectus sheath block, lower rest pain scores at 0–2 and 10–12 hours postoperatively were recorded compared to the control patients. In addition, the rectus sheath block significantly reduced pain scores of mobility at 0–2 hours postoperatively. Moreover, 24-hour opioid consumption and opioid-related side effects were also lower in the rectus sheath block compared to the control group [27].

In addition, comparative studies which examined the effect of TAP block versus subcostal TAP block have been published. Bhatia et al. conducted a prospective, randomized, double-blind study in which 60 patients who underwent LC received the standard general anesthesia or an ultrasound-guided posterior TAP or a subcostal TAP block. At the initial postoperative measurement times, the subcostal and posterior TAP groups had comparable pain scores. However, 4 hours after the operation, the scores were significantly lower in patients who had received the subcostal TAP block compared to those who were treated with posterior TAP [31]. In a prospective, randomized, double-blinded clinical study, Oksar et al. discovered that analgesic consumption after LC was greater among patients who were treated with controlled analgesia compared to patients who were treated with TAP or oblique subcostal TAP blocks [32].

However, as of yet, there have not been extensive studies of the effect of combined peripheral nerve block techniques in reducing LC postoperative pain scores and the reduction of the consumption of additional analgetic medications. The rationale underlying the combination of the blocks is that administration of both peripheral blocks, each affecting different nerve areas (TAP-T6-L1, subcostal TAP-T7-T12, and rectus sheath-T7-T11), could affect and block the entire area of surgery. Thus, the aim of the current study was to evaluate the postoperative pain control of a combination of TAP + subcostal TAP blocks and subcostal TAP + rectus sheath blocks compared to TAP or subcostal TAP blocks as a standalone treatment. The maximal level of pain upon LC is recorded during the first 24 hours after surgery [4]. Therefore, the time points in which the pain levels and the amount of analgesic medication consumption were examined in this study were 30 and 60 minutes, as well as 6, 12, and 24 hours after LC surgery. Our data revealed that although a decrease in the level of pain was detected over time in all the block type groups, there was no difference between the various block types groups at any time point with regard to pain severity. Nevertheless, a significant difference in favor of the combined blocks treatment was observed regarding the number of medications consumed by each of the patients. The patients who received the combined treatment required less medication than those who were administered a single block treatment.

Jung et al. examined the effect of the combination of subcostal and lateral TAP blocks on the quality of recovery among 38 patients who underwent LC compared to control patients who underwent a sham block. The primary outcome of the study was the score of the quality of recovery-40 (QoR-40) questionnaire. The combination of the two blocks did not improve the QoR-40 or analgesic consumption during the first 24 hours after the surgery [33]. A prospective, randomized control study was conducted by Ramkiran et al. which included 61 patients scheduled for LC. The patients were randomized into 3 groups: combined subcostal TAP block with rectus sheath block (*n* = 20), oblique subcostal TAP block alone, and a conventional port-site infiltration group as a control group (*n* = 20). The combined group as well as the oblique subcostal TAP block alone presented lower pain scores during the time points up to 24 hours postoperation, as well as the reduced use of analgesia compared to the control group [34]. Those studies supported our findings, that there was no difference in the effect on pain intensity. However, we did find a difference in the number of analgesic medications used, which was lower in the combined treatment compared to the standalone blocks.

The lack of difference in the pain level and consequent analgesic consumption between the various block combinations and the standalone blocks might be explained by the fact that the analgesia regimen administered to patients obscured the blocks effect. In addition, the various antinociceptive agents which are included in multimodal general anesthesia affect multiple neurotransmitters and neural relays and thus offset the supposed additive effects of the combined or the standalone blocks [35].

## 5. Conclusion

The results of the present study indicated that a combination of peripheral nerve blocks reduced the use of analgesic consumption during the 24 hours after LC surgery, compared to standalone blocks. Considering these findings, we recommend their use for reducing the administration of additional analgesics shortly after LC.

### 5.1. Limitations

The present study has some limitations. It was a single-center study with a small sample size. Pain assessments were not performed by a single caregiver, which could have led to a bias in the reporting scores. Also, the pain scores did not evaluate the patient's pain during movement. In addition, nausea and vomiting were not assessed.

## Figures and Tables

**Table 1:** Comparison of pain levels at any time point between the groups.

Time post-surgery	All patients	Block types				
		TAP	Subcostal TAP	Subcostal TAP + TAP	Subcostal TAP + Rectus Sheath	*P* value
30 min	3.6 ± 3.2	3.8 ± 3.1	4.0 ± 3.2	3.6 ± 3.3	2.9 ± 3.2	0.321
60 min	3.3 ± 3.0	2.7 ± 3.1	3.6 ± 3.1	2.8 ± 3.1	3.9 ± 2.8	0.131
6 hours	2.1 ± 2.6	1.8 ± 2.6	2.4 ± 2.6	1.8 ± 2.5	2.2 ± 2.7	0.585
12 hours	2.0 ± 2.6	1.8 ± 2.5	2.3 ± 2.8	1.6 ± 2.4	2.2 ± 2.5	0.497
24 hours	0.9 ± 2.0	1.1 ± 2.2	0.6 ± 1.8	0.7 ± 1.9	1.0 ± 2.1	0.462

Data are presented as mean ± standard deviation.

**Table 2:** Percentage of patients from all the groups who consumed analgesic medications over time.

Time post-surgery	Medication types % of patients							
	Paracetamol	Tramal	Morphine	Dipyrone IV	Ketorolac	Pethidine	Voltaren	Total
30 min	31	4	41	4	1	2		83
60 min	25	13	20	13				71
6 hours	11	13	1	13			1	39
12 hours	8	14	0	16			5	43
24 hours	6	5	0	8			2	21
Total	81	49	62	54	1	2	8	

Data are presented as % of patients who were treated with each of the medicine.

**Table 3:** Mean/median number of medications consumed by each of the patients from all treatment group.

Blocks	# Of medications per patient
TAP	
Mean	2.7
Median	3
Subcostal TAP	
Mean	2.8
Median	3
Subcostal TAP + TAP	
Mean	2.3
Median	2
Subcostal TAP + rectus sheath	
Mean	2.5
Median	2

**Table 4:** Mean/median number of medications consumed by each of the patients treated with a single blocks compared to combined blocks treatments.

Blocks	# Of medications
Single blocks	
TAP	
Subcostal TAP	
Mean	2.73
Median	3
Combined blocks	
Subcostal TAP + TAP	
Subcostal TAP + rectus sheath	
Mean	2.4
Median	2

Independent-samples Kolmogorov–Smirnov Test, Pv = 0.024.

## Data Availability

The data used in this study are available upon request and approval of the Shamir Medical Center.

## References

[1] Antoniou S. A., Antoniou G. A., Koch O. O., Pointner R., Granderath F. A. (2014). Meta-analysis of laparoscopic vs open cholecystectomy in elderly patients. *World Journal of Gastroenterology*.

[2] Wang W., Wang L., Gao Y. (2021). A meta-analysis of randomized controlled trials concerning the efficacy of transversus abdominis plane block for pain control after laparoscopic cholecystectomy. *Frontiers in surgery*.

[3] Nassar A. H. M., Zanati H. E., Ng H. J., Khan K. S., Wood C. (2022). Open conversion in laparoscopic cholecystectomy and bile duct exploration: subspecialisation safely reduces the conversion rates. *Surgical Endoscopy*.

[4] Rahimzadeh P., Faiz S. H. R., Latifi-Naibin K., Alimian M. A. (2022). A Comparison of effect of preemptive versus postoperative use of ultrasound-guided bilateral transversus abdominis plane (TAP) block on pain relief after laparoscopic cholecystectomy. *Scientific Reports*.

[5] Bisgaard T., Klarskov B., Rosenberg J., Kehlet H. (2001). Characteristics and prediction of early pain after laparoscopic cholecystectomy. *Pain*.

[6] Tsai H. W., Chen Y. J., Ho C. M. (2011). Maneuvers to decrease laparoscopy-induced shoulder and upper abdominal pain: a randomized controlled study. *Archives of Surgery*.

[7] Gerbershagen H. J., Aduckathil S., van Wijck A. J. M., Peelen L. M., Kalkman C. J., Meissner W. (2013). Pain intensity on the first day after surgery: a prospective cohort study comparing 179 surgical procedures. *Anesthesiology*.

[8] Slinger P. (2014). From the Journal archives: postoperative analgesia: effect on lung volumes. *Canadian Journal of Anesthesia/Journal canadien d’anesthésie*.

[9] Boddy P., Mehta S., Rhodes M. (2006). The effect of intraperitoneal local anesthesia in laparoscopic cholecystectomy: a systematic review and meta-analysis. *Anesthesia and Analgesia*.

[10] Hong D., Flood P., Diaz G. (2008). The side effects of morphine and hydromorphone patient-controlled analgesia. *Anesthesia and Analgesia*.

[11] Barazanchi A. W. H., MacFater W. S., Rahiri J. L. (2018). Evidence-based management of pain after laparoscopic cholecystectomy: a PROSPECT review update. *British Journal of Anaesthesia*.

[12] Rafi A. N. (2001). Abdominal field block: a new approach via the lumbar triangle. *Anaesthesia*.

[13] Johns N., O’Neill S., Ventham N. T., Barron F., Brady R. R., Daniel T. (2012). Clinical effectiveness of transversus abdominis plane (TAP) block in abdominal surgery: a systematic review and meta-analysis. *Colorectal Disease: The Official Journal of the Association of Coloproctology of Great Britain and Ireland*.

[14] Rozen W. M., Tran T. M. N., Ashton M. W., Barrington M. J., Ivanusic J. J., Taylor G. I. (2008). Refining the course of the thoracolumbar nerves: a new understanding of the innervation of the anterior abdominal wall. *Clinical Anatomy*.

[15] Tekeli A. E., Eker E., Bartin M. K., Öner M. O. (2020). The efficacy of transversus abdominis plane block for postoperative analgesia in laparoscopic cholecystectomy cases: a retrospective evaluation of 515 patients. *Journal of International Medical Research*.

[16] Champaneria R., Shah L., Geoghegan J., Gupta J. K., Daniels J. P. (2013). Analgesic effectiveness of transversus abdominis plane blocks after hysterectomy: a meta-analysis. *European Journal of Obstetrics and Gynecology and Reproductive Biology*.

[17] Elkassabany N., Ahmed M., Malkowicz S. B., Heitjan D. F., Isserman J. A., Ochroch E. A. (2013). Comparison between the analgesic efficacy of transversus abdominis plane (TAP) block and placebo in open retropubic radical prostatectomy: a prospective, randomized, double-blinded study. *Journal of Clinical Anesthesia*.

[18] Brogi E., Kazan R., Cyr S., Giunta F., Hemmerling T. M. (2016). Transversus abdominal plane block for postoperative analgesia: a systematic review and meta-analysis of randomized-controlled trials. *Canadian Journal of Anesthesia/Journal canadien d’anesthésie*.

[19] Jeffrey K. N., Thelen A. E., Dreimiller A. M. (2023). Laparoscopic transversus abdominis plane block reduces postoperative opioid requirements after laparoscopic cholecystectomy. *Surgery*.

[20] Çaparlar C. Ö., Özhan M. Ö., Süzer M. A. (2017). Fast-track anesthesia in patients undergoing outpatient laparoscopic cholecystectomy: comparison of sevoflurane with total intravenous anesthesia. *Journal of Clinical Anesthesia*.

[21] Hebbard P. D., Barrington M. J., Vasey C. (2010). Ultrasound-guided continuous oblique subcostal transversus abdominis plane blockade: description of anatomy and clinical technique. *Regional Anesthesia and Pain Medicine*.

[22] Wu L., Wu L., Sun H., Dong C., Yu J. (2019). Effect of ultrasound-guided peripheral nerve blocks of the abdominal wall on pain relief after laparoscopic cholecystectomy. *Journal of Pain Research*.

[23] Peng K., Ji F., Liu H., Wu S. (2016). Ultrasound-guided transversus abdominis plane block for analgesia in laparoscopic cholecystectomy: a systematic review and meta-analysis. *Medical Principles and Practice*.

[24] Emile S. H., Elfeki H., Elbahrawy K., Sakr A., Shalaby M. (2022). Ultrasound-guided versus laparoscopic-guided subcostal transversus abdominis plane (TAP) block versus No TAP block in laparoscopic cholecystectomy; a randomized double-blind controlled trial. *International Journal of Surgery*.

[25] Quek K. H. Y., Phua D. S. K. (2014). Bilateral rectus sheath blocks as the single anaesthetic technique for an open infraumbilical hernia repair. *Singapore Medical Journal*.

[26] Mavarez A. C., Ahmed A. A. (2023). Transabdominal plane block. https://www.ncbi.nlm.nih.gov/books/NBK560527/.

[27] Hamid H. K. S., Ahmed A. Y., Alhamo M. A., Davis G. N. (2021). Efficacy and safety profile of rectus sheath block in adult laparoscopic surgery: a meta-analysis. *Journal of Surgical Research*.

[28] Kadam V. R., Howell S., Kadam V. (2016). Evaluation of postoperative pain scores following ultrasound guided transversus abdominis plane block versus local infiltration following day surgery laparoscopic cholecystectomy-retrospective study. *Journal of Anaesthesiology Clinical Pharmacology*.

[29] Vindal A., Sarda H., Lal P. (2021). Laparoscopically guided transversus abdominis plane block offers better pain relief after laparoscopic cholecystectomy: results of a triple blind randomized controlled trial. *Surgical Endoscopy*.

[30] Tolchard S., Davies R., Martindale S. (2012). Efficacy of the subcostal transversus abdominis plane block in laparoscopic cholecystectomy: comparison with conventional port-site infiltration. *Journal of Anaesthesiology Clinical Pharmacology*.

[31] Bhatia N., Arora S., Jyotsna W., Kaur G. (2014). Comparison of posterior and subcostal approaches to ultrasound-guided transverse abdominis plane block for postoperative analgesia in laparoscopic cholecystectomy. *Journal of Clinical Anesthesia*.

[32] Oksar M., Koyuncu O., Turhanoglu S., Temiz M., Oran M. C. (2016). Transversus abdominis plane block as a component of multimodal analgesia for laparoscopic cholecystectomy. *Journal of Clinical Anesthesia*.

[33] Jung J., Jung W., Ko E. Y. (2021). Impact of bilateral subcostal plus lateral transversus abdominis plane block on quality of recovery after laparoscopic cholecystectomy: a randomized placebo-controlled trial. *Anesthesia and Analgesia*.

[34] Ramkiran S., Jacob M., Honwad M., Vivekanand D., Krishnakumar M., Patrikar S. (2018). Ultrasound-guided combined fascial plane blocks as an intervention for pain management after laparoscopic cholecystectomy: a randomized control study. *Anesthesia: Essays and Researches*.

[35] Brown E. N., Pavone K. J., Naranjo M. (2018). Multimodal general anesthesia: theory and practice. *Anesthesia and Analgesia*.

